# Integrated Transcriptional and Proteomic Profiling Reveals Potential Amino Acid Transporters Targeted by Nitrogen Limitation Adaptation

**DOI:** 10.3390/ijms21062171

**Published:** 2020-03-21

**Authors:** Qiong Liao, Tian-jiao Tang, Ting Zhou, Hai-xing Song, Ying-peng Hua, Zhen-hua Zhang

**Affiliations:** 1 Southern Regional Collaborative Innovation Center for Grain and Oil Crops in China, College of Resources and Environmental Sciences, Hunan Agricultural University, Changsha 430128, China; 18229474886@163.com (Q.L.); TTJ0514@163.com (T.-j.T.); hyp19890413@163.com (H.-x.S.); 2 School of Agricultural Sciences, Zhengzhou University, Zhengzhou 475000, China; zhoutt@zzu.edu.cn

**Keywords:** NLA, N limitation, transcriptional profiling, proteomic change, N remobilization, amino acid transporter

## Abstract

Nitrogen (N) is essential for plant growth and crop productivity. Organic N is a major form of remobilized N in plants’ response to N limitation. It is necessary to understand the regulatory role of N limitation adaption (NLA) in organic N remobilization for this adaptive response. Transcriptional and proteomic analyses were integrated to investigate differential responses of wild-type (WT) and *nla* mutant plants to N limitation and to identify the core organic N transporters targeted by NLA. Under N limitation, the *nla* mutant presented an early senescence with faster chlorophyll loss and less anthocyanin accumulation than the WT, and more N was transported out of the aging leaves in the form of amino acids. High-throughput transcriptomic and proteomic analyses revealed that N limitation repressed genes involved in photosynthesis and protein synthesis, and promoted proteolysis; these changes were higher in the *nla* mutant than in the WT. Both transcriptional and proteomic profiling demonstrated that LHT1, responsible for amino acid remobilization, were only significantly upregulated in the *nla* mutant under N limitation. These findings indicate that NLA might target LHT1 and regulate organic N remobilization, thereby improving our understanding of the regulatory role of NLA on N remobilization under N limitation.

## 1. Introduction

Nitrogen (N) is a macronutrient essential for plant growth and seed yield. To achieve a high seed yield, a large quantity of chemical N fertilizers is applied to crop fields. China is the largest N-consuming country in the world, accounting for up to one-third of the world’s N consumption [1]. However, the average N use efficiency (NUE) in China is approximately 30%, which is considerably less than that in other developed countries [2]. Excessive N application results in numerous environmental problems, including ecosystem saturation, global warming, and water pollution [3]. Exploiting the maximum potential of chemical fertilizers by improving the NUE is important for sustainable agriculture and environmental protection [4,5]. In order to adapt to variations in N availability in soils, plants have evolved multiple strategies, which include different N uptake systems and N remobilization.

Nitrate (NO_3_^−^) is the main N source for upland plants, and it also functions as a signal molecule [6]. Four NO_3_^−^ transporters, namely NO_3_^−^ transporter 1 and 2 (NRT1 and NRT2), chloride channel (CLC), and slow-type anion channel (SLAC1/SLAH), have been reported to be responsible for NO_3_^−^ uptake and remobilization [7]. Once absorbed by the roots, NO_3_^−^ can be either stored in the root vacuole or assimilated into amino acids via the nitrate reductase (NR)-glutamine synthetase (GS)/glutamate synthase (GOGAT) pathway; however, most NO_3_^−^ can be loaded into xylem vessels and transported to the shoot, and this process is essential for plant growth [8]. NO_3_^−^ remobilization in plants involves both short- and long-distance distribution. The *CLC* gene family regulates short-distance NO_3_^−^ distribution between the cytoplasm and vacuole. In *Arabidopsis thaliana*, AtCLCa was initially identified as a 2NO_3_^−^/1H^+^ antiporter and the NO_3_^−^ content was reduced by approximately 50% in the *Atclca* mutant [9], although NUE was shown to increase significantly in the *Atclca*-2 mutant compared with the wild-type (WT) plants [8,10]. Long-distance NO_3_^−^ distribution is coordinated and regulated by members of the *NRT1* gene family, and, in *A. thaliana*, *AtNRT1*.5/*AtNPF7*.3, which is mainly expressed in root pericycle cells near the xylem, is responsible for NO_3_^−^ loading. Consistently, less NO_3_^−^ was detected in the xylem sap and shoot of the *nrt1*.*5* mutant [11]. In contrast to *AtNRT1*.5/*AtNPF7*.*3*, *AtNRT1.8/AtNPF7.2*, which is mainly expressed in xylem parenchyma cells in the vasculature, participates in NO_3_^−^ unloading; accordingly, a higher NO_3_^−^ shoot/root (S/R) ratio was observed in the *nrt1.8* mutant [12]. Thus, *AtNRT1*.*5* and *AtNRT1*.*8* control the proportion of NO_3_^−^ distributed to the shoot and play important roles in improving the NUE of plants [8]. The gene pair *AtNRT1.7/AtNPF2.13* is expressed in parenchyma cells of leaf phloem, and it regulates NO_3_^−^ remobilization from old to young leaves [13].

The N limitation adaption (NLA) protein, a RING-type E3 ubiquitin ligase, regulates proteolysis, and it is essential for maintaining nutrient homeostasis in plants [14,15]. This protein was first isolated from an *A. thaliana* mutant that failed to develop essential adaptive responses to N limitation [16]. Additionally, NLA is involved in the accumulation of salicylic acid and responsible for immune responses [17]. It plays an important role in the regulation of inorganic phosphate (Pi) homeostasis, but not N homeostasis, as early senescence in *nla* mutants was caused by Pi toxicity [18]. However, Liu et al. [19] observed that the ^15^NO_3_^−^ isotope spotted on old leaves preferentially accumulated in the youngest leaves of the *nla* mutant. Under N-limited conditions, NLA transcripts remained unchanged; however, the NLA protein abundance substantially decreased after N limitation, indicating that regulation occurs mainly at the translational level. It was also found that *NRT1*.*7* can be degraded by NLA, which regulates NO_3_^−^ remobilization from sources to sinks via the ubiquitin-mediated post-translational regulatory pathway [19]. Moreover, the genes involved in proteolysis, N transportation, and anthocyanin synthesis were upregulated in the *nla* mutant [20]. However, the fundamental function of NLA in the regulation of N homeostasis requires further investigation.

Although NO_3_^−^ remobilization regulated by the miR827-NLA-NRT1.7 pathway certainly contributes to the source to sink distribution, NO_3_^−^ generally remains at a low level (relative to the total N) in plants [21]. Up to 75% of the absorbed N is stored in chloroplastic proteins and in ribulose-1,5-bisphosphate carboxylase/oxygenase [22]. Although proteolysis and amino acid remobilization are likely to be predominant in N remobilization [7,22], it remains unclear which amino acid transporters can be poly-ubiquitinated by NLA. It has been shown that NLA interacts with ORE1 (ANAC092) in the nucleus and regulates its stability through poly-ubiquitination, which determines leaf senescence during N deficiency [23]. These findings suggest that the ubiquitination degradation substrate of NLA is not specific [19].

In the present study, we (i) examined the different physiological responses of WT and *nla* mutant plants to N limitation, (ii) conducted transcriptional and proteomic profiling to reveal the molecular strategies of N limitation adaptation, and (iii) proposed a core targeting substrate for NLA, which is involved in the efficient remobilization of organic N nutrients. Thus, the present results will provide baseline information on efficient N remobilization from sources to sinks in response to N limitation that can be used for the genetic improvement of NUE in plants.

## 2. Results

### 2.1. Differential Physiological Responses of the WT and nla Mutant Plants to N Limitation

**Figure 1:**
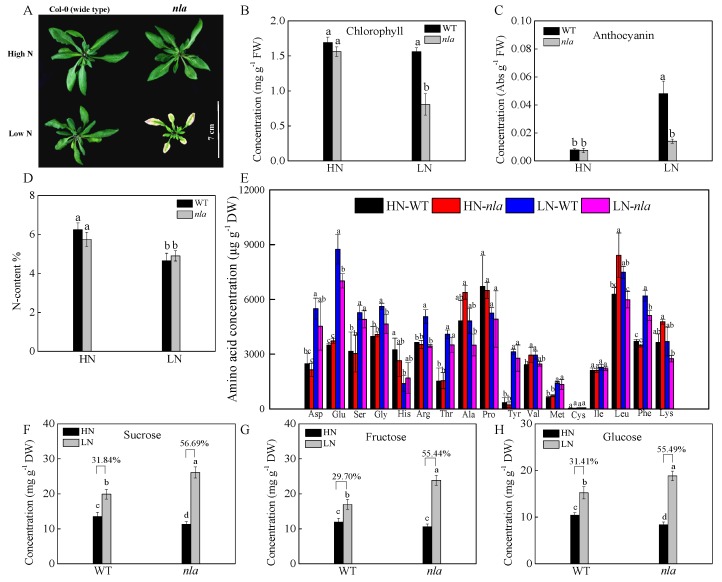
Physiological responses to N limitation in the wild-type (WT) and N limitation adaption (*nla*) mutant. The WT and *nla* mutant plants were grown hydroponically under 4.5 mM NO_3_^−^ (HN) for 10 d and then exposed to 0.3 mM NO_3_^−^ (LN) for 3 d, the rosette leaves were sampled for the following assays. (**A**) The phenotype of the WT and *nla* under HN and LN conditions, bar scale = 7 cm, (**B**) leaf chlorophyll concentration, (**C**) leaf anthocyanin concentration, (**D**) N content (N%) of the whole plants, (**E**) amino acid concentration in the leaves, (**F**–**H**) concentrations of sucrose (**F**), fructose (**G**), and glucose (**H**) in leaves. The presented data are the means ± SE of three independent biological replicates. The different letters at the top of the histogram bars denote significant differences at *p* < 0.05.

We investigated the physiological responses of the WT and *nla* mutant plants grown hydroponically under high (HN, 4.5 mM) and low (LN, 0.30 mM) NO_3_^−^ conditions. After 10 d of growth under HN, the plants were transferred to the LN for 3 d. Under N-sufficient conditions, the WT and *nla* mutant performed similarly, showing similar chlorophyll and anthocyanin content (Figure 1A–C). Nitrogen limitation resulted in decreased chlorophyll content and induced anthocyanin accumulation in both WT and *nla* mutant (Figure 1B,C). However, compared with the WT, the *nla* mutant showed lower chlorophyll and anthocyanin content (Figure 1B,C) and presented hypersensitivity to N limitation (Figure 1A). Although the *nla* mutant displayed an early senescence phenotype induced by N limitation, the total N absorbed by these plants was not significantly different from that absorbed by the WT under both HN and LN conditions (Figure 1D). 

To survive under N-limited conditions, plants tend to remobilize N from old leaves to young organs [24]. We further investigated the content of 17 amino acids in the WT and *nla* mutant plants under the two contrast N conditions. Under the N-sufficient conditions, no significant difference was observed between the WT and *nla* mutant, but N limitation increased amino acids in both the WT and *nla* mutant, probably via the induction of protein degradation. Moreover, the WT plants had more amino acids in the rosette leaves than the *nla* mutant plants (Figure 1E). Low N availability restricted amino acid biosynthesis, which resulted in an increase in organic acids available for sugar synthesis [25,26]. Sucrose, fructose, and glucose accumulated considerably in the WT under N-limited conditions, increasing by 31.84%, 29.70%, and 31.41%, respectively (Figure 1F–H). However, when compared with WT, sucrose, fructose, and glucose in the *nla* mutant increased by 56.69%, 55.44%, and 55.49% under N-limited conditions, respectively. The *nla* mutant accumulated more sugar than the WT under N limitation, which might be due to lower N availability initially. These data suggested that the *nla* mutant was more sensitive to N limitation, as indicated by early leaf senescence.

### 2.2. Transcriptional Profiling Reveals Different Responses to N Limitation between the WT and nla Mutant Plants 

The *nla* mutant showed greater hypersensitivity to N limitation than the WT (Figure 1). To further examine this discrepancy, we compared the transcript abundance of the N limitation-repressed genes involved in photosynthesis, protein synthesis, and degradation [20]. Nitrogen limitation repressed all genes involved in photosynthesis in both WT and *nla* mutant, and 10 consistently repressed genes are listed in Table 1. Compared with N-sufficient conditions, the expression of these 10 genes decreased by 1.9- to 3.74-fold and 2.75- to 8.01-fold in the WT and *nla* mutant under N-limited conditions, respectively (Table 1). Nitrogen is a critical component for protein synthesis, and protein synthesis will likely be constrained by N limitation [24]. Indeed, N limitation decreased the expression of all genes involved in protein synthesis in both WT and *nla* mutant [20]; 16 consistently repressed genes are listed in Table 1. Compared with N-sufficient conditions, the expression of these 16 genes decreased by 1.99- to 4.87-fold and 3.16- to 7.45-fold in the WT and *nla* mutant under N-limited conditions, respectively (Table 1). 

To survive under N-limited conditions, plants tend to degrade proteins in mature leaves and export the resulting amino acids to young organs [24]. We compared the fold-change in proteolytic-associated genes induced by N limitation in both WT and *nla* mutant; our results revealed changes that ranged from 2.20- to 13.36-fold in the *nla* mutant, which was considerably higher than that in the WT (2.04- to 7.48-fold). Moreover, compared with N-sufficient conditions, nine genes responsible for N uptake and transport were significantly upregulated by 2.07- to 18.65-fold in the *nla* mutant under N limited conditions, but showed no significant change in the WT (Table 2). Specifically, *AtLHT1* and *AtLHT7*, responsible for lysine and histidine transport, were significantly upregulated by more than 15-fold. Additionally, *AtCAT1*, a cationic amino acid transporter, was upregulated by 6.21-fold, and *AtPUT2/AtPQR2*, belonging to the amino acid permease family, was upregulated by 2.93-fold. The expression of *AtAAP4* and *AtAAP1* was upregulated by 2-fold. In addition to the genes involved in amino acid transport, *AtAMT1.1* and *AtAMT2*, responsible for N uptake, were significantly upregulated in the *nla* mutant under N limited conditions. These data suggested that the mutation in *NLA* results in severe protein degradation and rapid N remobilization under N-limited conditions, which led, partially, to the early senescence of the *nla* mutant.

Previous studies have demonstrated that N limitation induces anthocyanin over-accumulation [20]. Many genes, including *PAL*, *CHS*, *F3R*, *DFR*, *CL3*, and *ANS*, regulate anthocyanin formation, and most of them were upregulated by N limitation in both WT and *nla* mutant plants (Table 2). Additionally, *MYB*, a transcription factor, is reported to play a negative role in the regulation of anthocyanin synthesis under N limitation [27,28,29]. The changes in the expression of four *MYB* transcription factors between the two N conditions were investigated. *MYB90* was upregulated by 29.11- and 35.98-fold in the WT and *nla* mutant, respectively; N limitation specifically induced *MYB2* by 24.55-fold in the *nla* mutant, whereas the expression of *MYB75* and *MYB32* was upregulated in the WT under N-limited conditions. Despite the significant upregulation of the genes responsible for anthocyanin production, the *nla* mutant failed to accumulate anthocyanin and acclimate to N limitation, presenting early leaf senescence (Figure 1). The transcriptomic profiling showed that senescence-associated genes were significantly upregulated in the *nla* mutant (Table 2).

### 2.3. iTRAQ Data Analysis and Protein Identification in the nla Mutant and WT under N-Sufficient and N-Limited Conditions

The NLA protein is a RING-type E3 ubiquitin-ligase responsible for proteolysis [14,15]. To further investigate protein changes in the *nla* mutant under N limitation, proteomic sequencing based on the iTRAQ technology was performed. A total of 237,353 spectra, 38,790 peptides, and 30,329 unique peptides were identified (Figure S1A). Furthermore, 6513 proteins were detected by at least one unique peptide (Figure S1A). The molecular weight of the identified proteins ranged from 1.74 to 609.09 kDa (Figure S1B), where proteins of 20–30 and 30–40 kDa were the most abundant, followed by proteins of 40–50, 10–20, and 50–60 kDa (Figure S1B). The protein number decreased with the coverage of the molecular weight. The isoelectric points of the identified proteins were 5–10 (Figure S1C). Most of the identified proteins contained less than 10 peptides, and fewer proteins were detected as the number of peptides increased (Figure S1D). To evaluate the reliability of the protein quantification data, the peptide ion scores were calculated. Over 78% of the identified proteins had a score of more than 20 points and the median score was greater than 33, which indicated that the iTRAQ data were reliable (Figure S2).

**Figure 2:**
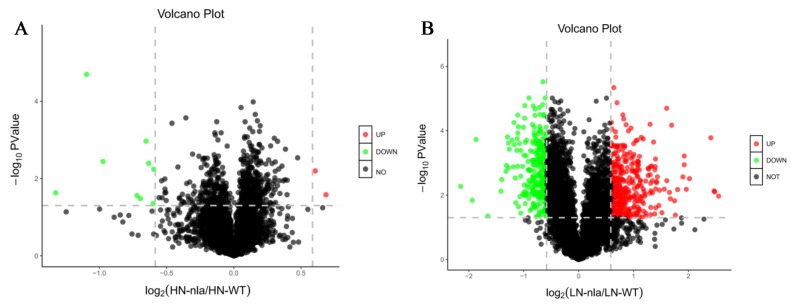
Differentially expressed proteins (DEPs) of *nla* vs. WT under N-sufficient and N-limited conditions. (**A**) DEPs of *nla* vs. WT plants under N-sufficient conditions. (**B**) DEPs of *nla* vs. WT plants under N-limited conditions. The circles in the volcano plot represent the DEPs, the black ones indicate the DEPs with no significance, the red ones indicate significantly upregulated DEPs, and the green ones indicate significantly downregulated DEPs. The dash line indicate the fold change equal to 1.5.

To investigate the proteins responsive to N limitation, we performed a volcano plot analysis (Figure 2) using the fold-change and *p*-values from pairwise comparisons between the *nla* mutant and WT under N-limited conditions. The black dots indicate the proteins with no significant change, the red dots represent the DEPs with a fold-change of >1.5 and *p* < 0.05, and the green dots represent the DEPs with a fold-change of <0.7 and *p* < 0.05. Under N-sufficient conditions, most proteins showed no significant change between the *nla* mutant and WT, and only 11 proteins were identified as DEPs. Among them, two DEPs were significantly upregulated whereas nine DEPs were significantly downregulated (Figure 2A). However, 618 proteins exhibited significant changes in the *nla* mutant under N-limited conditions when compared with the WT (Figure 2B), suggesting that N limitation significantly altered protein expression in the *nla* mutant. Specifically, 347 proteins were significantly upregulated and 271 were downregulated in the *nla* mutant compared with the WT under N-limited conditions (Figure 2B).

### 2.4. Functional Analysis of the DEPs in Response to N Limitation

**Figure 3:**
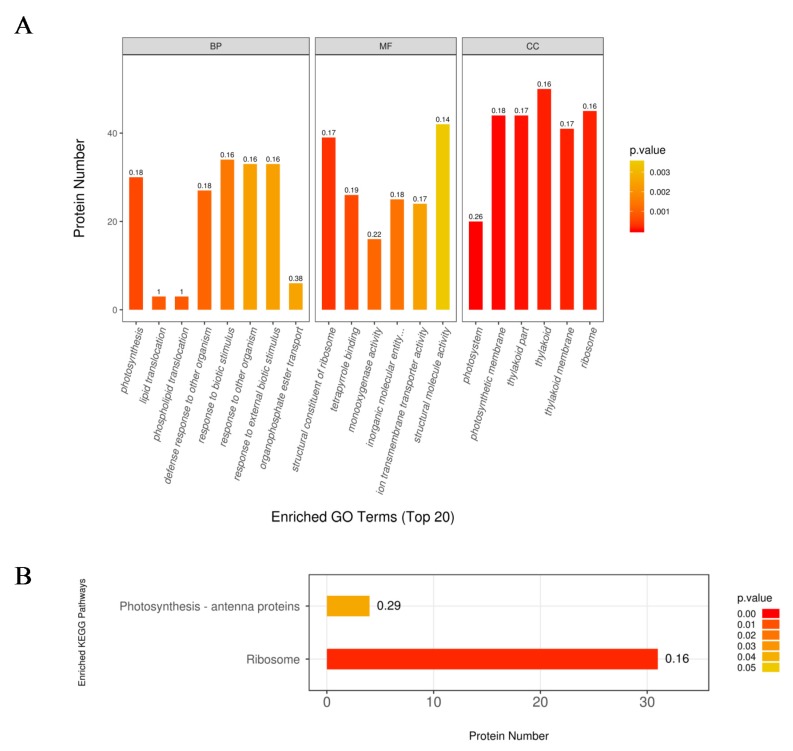
The gene ontology (GO) (**A**) and Kyoto Encyclopedia of Genes and Genomes (KEGG) (**B**) enrichment analysis of the upregulated proteins in the *nla* vs. WT comparisn under the N-limited conditions. The numbers above the bar-plot are rich factors, which represent the proportion of the DEPs relative to the total proteins identified in the specific pathway.

The 618 DEPs were classified into three major categories based on gene ontology (GO) enrichment analysis, namely, biological process, molecular function, and cellular component (Figure 3A). In the biological process category, the response to biotic stimulus process was the most represented term with 34 DEPs, followed by the response to other organism process (42.06%) and response to external biotic stimulus (Figure 3A). In addition, there were 30 and 27 DEPs enriched in the photosynthesis and defense response to other organism process, respectively. Structural molecule activity and structural constituent of ribosome were the two most enriched terms under the molecular function category, with 785 (45.48%) and 778 (45.08%) DEPs, respectively (Figure 3A). In the cellular component category, most DEPs were involved in thylakoid, ribosome, photosynthetic membrane, thylakoid part (30.48%), and thylakoid membrane (Figure 3A).

To further investigate key metabolic pathways that the DEPs expressed under N limitation are involved in, the Kyoto Encyclopedia of Genes and Genomes (KEGG) pathway enrichment analysis was performed with the 816 DEPs expressed under N-limited conditions. The results showed that the 618 DEPs were enriched in 73 metabolic pathways. Additionally, two pathways were highly enriched, the ribosome pathway was the most represented with approximately 31 DEPs (Figure 3B), and followed by photosynthesis antenna with four DEPs (Figure 3B).

### 2.5. N Limitation Represses Proteins Responsible for Photosynthesis and Protein Synthesis and Induced Proteins Related to Proteolysis and N Transport

Transcriptional analysis has revealed that N limitation repressed the genes involving in photosynthesis, protein synthesis, but induced the genes involved in protein degradation and N transport. We also investigated the response of these proteins responding to N limitation. As shown in Table 3, the DEPs involving in photosynthesis were downregulated by 1.51- to 2.46-fold under N limitation. Moreover, two DEPs contributing to chlorophyll biosynthesis were significantly repressed by N limitation, suggesting that N limitation severely affected the function of these proteins in photosynthesis (Table 3). Moreover, the DEPs related to protein synthesis and folding were downregulated by 1.51- to 1.90-fold under N limitation. Additionally, proteolysis-associated proteins were significantly upregulated, whereas the proteins involved in the ubiquitination pathway were repressed, likely due to the mutation in NLA (Table 4). The proteomic sequencing results showed that the nitrate transporters NRT1 and NAR2.1/NRT3.1 were upregulated by 1.81- and 2.85-fold, respectively, under N limitation. Two key enzymes, nitrate reductase and nitrite reductase, were significantly upregulated by N limitation, whereas GS, GDH1, and GDH2 were downregulated (Table 4). Additionally, LHT1, responsible for amino acid transport, was upregulated by 2.09-fold under N limitation (Table 4).

### 2.6. LHT1, Responsible for Amino Acid Transport, Was the Sole Gene Identified Both in Transcriptional and Proteomic Profiling

Both the transcriptional and proteomic analysis revealed that N limitation repressed the genes responsible for photosynthesis and protein synthesis and induced genes related to proteolysis and N transport (Table 1, Table 2, Table 3 and Table 4). In order to further investigate the identical genes identified both in transcriptional and proteomic analysis under N limitation, we listed the consistent genes in Table 5. The results showed that there were 49 genes identified both in transcriptional and proteomic analysis, which can be classified into four categories: (i) the genes both upregulated in transcriptome and proteomics analysis, (ii) the genes both downregulated in transcriptome and proteomics analysis, (iii) the genes upregulated in transcriptome but downregulated in proteomics analysis, and (iv) the genes downregulated in transcriptome but upregulated in proteomics analysis. The four categories accounted for 57.14%, 34.69%, 6.12%, and 2.04% of the total genes, respectively (Table 5). The fold change of nine proteins were significantly upregulated by more than 2-fold. When investigating the fold changes of the nine genes in the transcriptional data, we observed that the fold changes of AT2G43510, At4g16260, AT5G40780, and AT1G02920 were significantly upregulated by 10-fold. Among the four genes, we observed that LHT1 (AT5G40780) was the sole gene that was responsible for amino acid transport identified in transcriptional and proteomic profiling.

### 2.7. Transcriptional Expression Patterns of Genes Regulated by NLA under N Limitation

**Figure 4:**
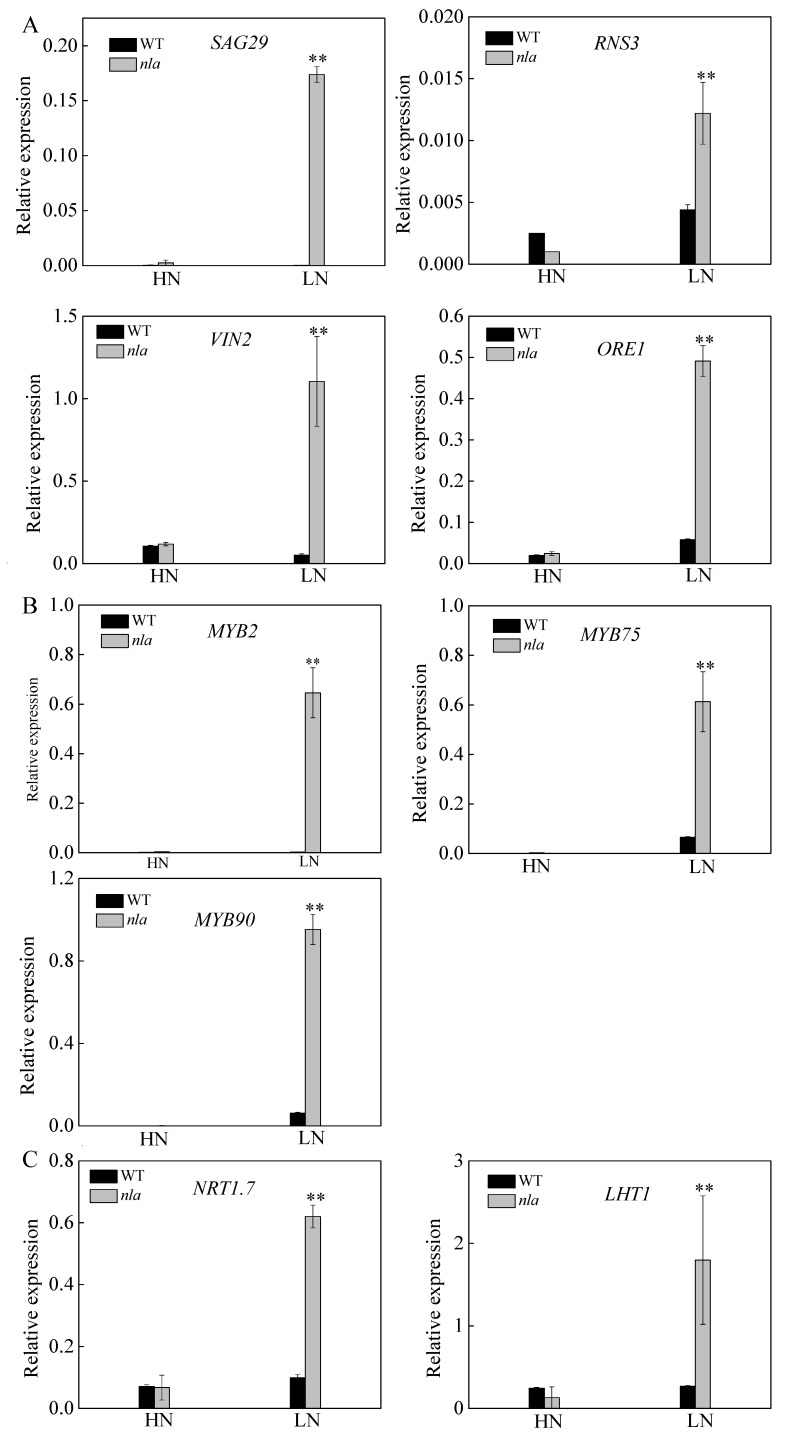
Relative expression of the genes involved in leaf senescence, MYB family, and N transport in the WT and *nla* mutant responding to N limitation. Relative expression levels of senescence-associated genes (**A**), *MYB* transcription factors (**B**), genes involved in N transport (**C**), as revealed by the q-PCR assays. For the N-limited treatment, the Arabidopsis seedlings were first grown under 4.5 mM NO_3_^−^ for 10 d, and then transferred to a solution containing 0.3 mM NO_3_^−^ for 3 d. Bars indicate the standard deviation (SD) of three biological replicates, **p < 0.01.

The *nla* mutant was more sensitive to N limitation than the WT, showing an early senescence phenotype (Figure 1). It is reported that *RNS3*, *SAG29*, and *VIN2* play important roles in the regulation of leaf senescence [30,31,32]. Moreover, ORE1 is reported to act downstream of NLA, contributing to the regulation of leaf senescence [23]. To reveal further differences in the expression of the genes encoding these proteins between the WT and *nla* mutant, we conducted the transcriptional analysis. The expression of these genes was considerably increased in the *nla* mutant under N limitation only (Figure 4A). In addition, N limitation significantly induced the expression of genes involved in anthocyanin production (Table 2). The expression of *MYB2*, *MYB75*, and *MYB90* was upregulated in both WT and *nla* mutant under N limitation; however, the transcripts of these genes were considerably higher in the *nla* mutant (Figure 4B). NRT1.7 can be degraded by NLA and mediates NO_3_^−^ remobilization from the sinks to source tissues [19]. Under N limitation, the relative expression of *NRT1.7* significantly increased in the *nla* mutant (Figure 4C). Moreover, the transcriptomic and proteomic profiling analyses revealed that *LHT1* was significantly upregulated in the *nla* mutant under N limitation (Table 2 and Table 4). We also observed that the relative expression of *LHT1* considerably increased in the *nla* mutant under N limitation (Figure 4C), suggesting that LHT1 might be targeted by NLA and mediate N remobilization.

## 3. Discussion

### 3.1. Differential Physiological and Molecular Responses of WT and nla Mutant Plants under N Limitation

N availability plays an important role in the regulation of plant growth and productivity. Under N limitation in the soil, plants evolve adaptive responses to cope with the fluctuating environment [33]. Physiological adaptive responses to N limitation include the reduction of plant growth and photosynthesis (Figure 1A,B). Approximately 80% of the total leaf N is stored in the chloroplasts mainly in the form of proteins, which are an important N pool for N remobilization [34]. The chlorophyll content was decreased both in the WT and *nla* mutant plants while acclimating to N limitation (Figure 1B). Moreover, all of the genes involved in chlorophyll formation and photosynthesis were downregulated by N limitation [20], the fold-change of which was higher in the *nla* mutant (Table 1 and Table 3), indicating it was more sensitive to N limitation. Furthermore, N limitation induced anthocyanin accumulation, which acts as a light-protecting pigment for N-deficient plants, preventing photo inhibition damage caused by N limitation [35,36]. While the WT plants accumulated a large quantity of anthocyanin under N limitation, the *nla* mutant lacked this adaptive response (Figure 1C). Transcription profiling analysis revealed that genes responsible for anthocyanin synthesis were upregulated under N limitation in both the WT and the *nla* mutant (Table 2), although there were few changes in anthocyanin accumulation in the *nla* mutant under N limitation (Figure 1C). The *MYB* transcription factors, particularly *MYB75* and *MYB90*, play a positive role in the regulation of anthocyanin synthesis [27,28,29], which were markedly induced by N limitation in the WT, while *MYB90* was upregulated in the *nla* mutant (Table 2). *MYB2*, a transcriptional repressor, functions in the negative regulation of anthocyanin biosynthesis [37] and it increased approximately 24-fold under N limitation in the *nla* mutant (Table 2). The relative expression level of *MYB2* also increased markedly in the *nla* mutant under N limitation (Figure 4B). Thus, *MYB2* might be targeted by NLA and play a key role in the regulation of anthocyanin biosynthesis in response to N limitation.

Despite of the lack of anthocyanin accumulation under N limitation, the *nla* mutant showed early senescence of the rosette leaves compared with the WT (Figure 1A). Leaf senescence is a genetically programmed developmental process that is regulated by the senescence-associated genes [38]. Several senescence-associated genes were upregulated in the *nla* mutant under N limitation only (Table 2), which was consistent with the q-PCR results (Figure 4A). Moreover, leaf senescence is associated with protein degradation, which is an adaptive response to N limitation [21]. Genes and enzymes involved in protein degradation were upregulated by N limitation in the WT and *nla* mutant, leading to an increase in the free amino acid content (Figure 1E). However, the fold change of these genes was much higher in the *nla* mutant (Table 2 and Table 4), indicating that more sequestered N was released from source tissues via protease activities during leaf senescence in the *nla* mutant. However, the *nla* mutant had a lower amino acid content than the WT under N limitation, which might be due to the excessive remobilization of the amino acid. Transcriptomic data revealed that six genes coding for amino acid transporters were markedly increased in the *nla* mutant (Table 2). These data suggested excessive proteolysis and N remobilization in the *nla* mutant, leading to its early senescence.

### 3.2. Amino Acid Transporters May Be Involved in Efficient N Remobilization Mediated by NLA

N is an essential plant macronutrient and its starvation induces early leaf senescence [39,40,41]. The NLA protein is an E3 ubiquitin ligase and the *nla* mutant was hypersensitive to N starvation. In a previous study, most of the ^15^NO_3_^−^ spotted in old leaves was preferentially allocated to the youngest leaves [19]. Moreover, NLA degrades NRT1.7 through the ubiquitin-mediated post-translational regulatory pathway and facilitates the remobilization of NO_3_^−^ from source to sink when adapting to limited N supply [19]. Our results also demonstrated that the expression of NRT1.7 was significantly higher in the *nla* mutant under N limitation (Figure 4C). The increase in NRT1.7 expression would enhance NO_3_^−^ remobilization from sources to sinks as an adaptive response to N limitation. However, the majority of N nutrients are exported from source leaves as amino acids instead of NO_3_^−^ during leaf senescence [7,21,38].

In *A. thaliana*, over 100 putative amino acid transporters have been identified in the amino acid-polyamine-choline (APC) transporter superfamily and as members of the usually multiple acids move in and out transporters (UmamiT) family [42,43]. Three families, including the amino acid permeases (AAPs), lysine/histidine-like transporters (LHTs), and proline and glycine betaine transporters (ProTs), belong to the APC group [7]. Several genes encoding amino acid transporters, including LHT1, LHT7, CAT1, PUT2, AAP4, and AAP1, were significantly upregulated in the *nla* mutant (Table 2), which resulted in lower amino acid content in the rosette leaf of the *nla* mutant under N limitation compared to that of the WT (Figure 1E). Additionally, the proteomic data revealed that LHT1 was significantly upregulated in the *nla* mutant in response to N limitation (Table 4). There were 49 identical genes observed both in transcriptional and proteomic analysis, and most of the genes showed the same expression patterns (Table 5). However, 6.12% of the total genes were upregulated in the transcriptome data whereas downregulated in the proteomics data. This might result from transcriptional and post-transcriptional regulation, such as alternative splicing and miRNA regulation [44,45,46]. On the other hand, about 2.63% of the total genes were downregulated in transcriptome but upregulated in proteomics analysis, which was putatively due to the translation control mechanism [47]. Among the 49 identical genes, LHT1 was the sole gene that was responsible for amino acid transport. Under N-limited condition, LHT1 was upregulated by 18.65- and 2.09-fold in the transcriptional and proteomic analysis, respectively (Table 5). LHT1 belongs to the LHTs family and it is expressed in both the rhizodermis and mesophyll of *A. thaliana* [48]. In addition, LHT1 imports neutral and acidic amino acids into the root and mediates amino acid transportation into leaf cells [48,49,50,51]. We further characterized its expression level in the *nla* mutant; similar to NRT1.7, the expression level of LHT1 was significantly higher in the *nla* mutant than in the WT under N limitation (Figure 4C). Therefore, NLA might target the amino acid transporters, particularly LHT1, to regulate organic N remobilization under N limitation. As an E3 ubiquitin ligase, NLA regulates the protein stability of NRT1.7 and ORE1 during acclimation to N deficiency [19,23].

**Figure 5:**
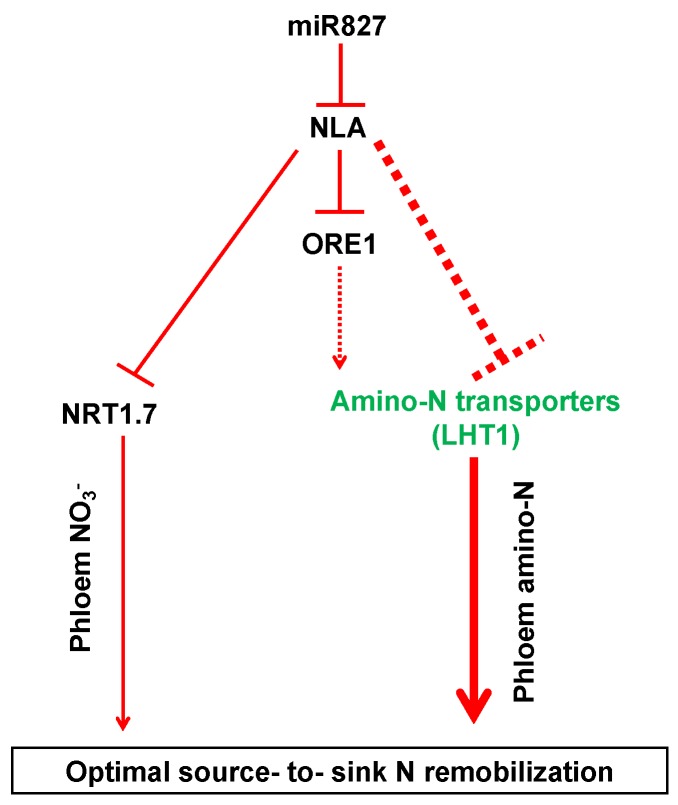
A proposed model for the adaptive strategies regulated by NLA responsive to N limitation. The dashed lines indicate potential or indirect regulation. The thicker the lines, the more important the pathway.

Based on the results obtained in the present study, we propose the following model: miR827 suppresses the expression of NLA under N limitation; conversely, NRT1.7 is induced to promote NO_3_^−^ remobilization from source tissues to sinks, while some amino acid transporters, particularly LHT1, are upregulated to enhance organic N remobilization from sources to sinks, thereby leading to the predominant N remobilization during adaptation to N limitation. Moreover, *ORE1*, a transcription factor gene acting downstream of NLA, might transcriptionally regulate amino acid transporters and contribute to the adaptive responses in plants under N deficiency (Figure 5). Together, our results provide comprehensive insights into the regulation of amino acid transporters by NLA and their contributions to organic N remobilization from sources to sinks in plants under N limitation.

## 4. Materials and Methods

### 4.1. Plant Materials and Growth Conditions

*Arabidopsis thaliana* ecotype Columbia (Col-0) was used as the control for the *nla* mutant. The *nla* mutant was provided by Dr. Wenxue Li (National Key Facility for Crop Gene Resources and Genetic Improvement, Institute of Crop Science, Chinese Academy of Agricultural Sciences, Beijing, China). The *nla* mutant was first screened and identified by Peng et al. [16]. The plant seeds were sown in a matrix containing vermiculite and perlite at a ratio of 3:2. After germination, the seedlings were transplanted into plastic boxes (5 L) with nutrient solution as described by Han et al. [8] and the nutrient solution (pH 5.7) was replenished every 5 d. The plants were hydroponically grown in an incubator according to a completely randomized block design with three biological replicates. The culture regime was set as follows: 70% humidity, 300–320 μmol m^−2^ s^−1^ illumination with a 16-h light/8-h dark cycle, and the temperature was set to 22 °C.

### 4.2. Determination of Chlorophyll, Anthocyanin, and N Content

After 10 d of hydroponic growth under 4.5 mM NO_3_^−^, the Col-0 and *nla* mutant plants were transferred to a nutrient solution with 0.3 mM NO_3_^−^ to simulate N-limited condition, and the rosette leaf and root were sampled individually 3 d later. Chlorophyll was extracted from *A. thaliana* leaf samples (0.15 g) by placing them in a tube with 10 mL of 1:1 absolute ethanol:acetone for 24 h; the absorbance of the solution was measured at 663, 645, and 652 nm. Leaf anthocyanin content was determined in the Col-0 and *nla* mutant according to the method described by Mancinelli et al. [52]. For N content determination, both shoot and root were oven dried at 105 °C for 30 min, and then at 65 °C until they reached a constant weight. Nitrogen content was determined using the Kjeldahl method [53].

### 4.3. Free Amino Acid Determination

The content of free amino acids was determined as described previously [54], with slight modifications. Specifically, approximately 0.1 g of leaf sample was added into a tube containing 1 mL of 80% ethanol, and the tube was placed in a water bath for 20 min at 80 °C. This procedure was repeated twice, and the extract was placed in an oven at 80 °C until the ethanol was completely evaporated. Then, 1.0 mL of 5.0 M NaOH was added to dissolve the sediment, which was then centrifuged at 12,000× *g* for 15 min. The resulting supernatant was collected and filtered through a membrane (2 mL); each filtrate (0.8 mL) was analyzed by high-performance liquid chromatography (HPLC) in the L-8800 HITACHI amino acid analyzer.

### 4.4. Determination of Sucrose, Fructose, and Glucose Content

The content of sucrose, fructose, and glucose was measured as described previously [55], with slight modifications. Leaf samples (1.0 g) were ground to powder with liquid N, and 5 mL of 80% ethanol was then added before placing the samples in a water bath for 30 min at 80 °C. This procedure was repeated twice, and the samples were centrifuged at 12,000× *g* for 20 min. The extracts were placed in an oven at 90 °C until the ethanol completely evaporated. Double distilled water (5 mL) was added to dissolve the sediment, and the resulting supernatant was collected and filtered through a membrane (0.45 μm) and analyzed by HPLC using the L-8800 HITACHI amino acid analyzer.

### 4.5. Transcriptional Responses of nla to N Limitation

To identify genome-wide mRNA transcriptomic responses of the *nla* mutant and Col-0 (WT) plants to N limitation, we retrieved the RNA-sequencing data reported by Peng et al. [20]. The plants were initially grown in nutrient-free soil, and then supplied with a nutrient solution containing 3 mM (low nitrogen, LN) or 10 mM (high nitrogen, HN) KNO_3_ once a week for 4 weeks. After 17, 21, and 25 d post-germination, the total RNA was extracted from the 5th–8th rosette leaves and used in the cDNA synthesis microarray hybridization analysis, as described in Zhu et al. [56]. We used the microarray data to compare the molecular responses induced by N limitation between the WT and *nla* mutant plants.

### 4.6. Protein Extraction, Digestion, and Isobaric Tags for Relative and Absolute Quantitation (iTRAQ) Labeling

Protein extraction was performed as previously described [57,58]. Briefly, plant samples were homogenized to fine powder in liquid N, and 0.1 g of each sample was then transferred into a 2-mL Eppendorf tube with three biological replicates. Each sample was homogenized with 4% (*w/v*) SDS, 100 mM Tris-HCl (pH 7.6), and 0.1 M DTT. The SDS-Tris-HCl-DTT (SDT) method was conducted for protein extraction and the bicinchoninic acid (BCA) method was used to quantify the protein [59]. Filter-aided sample preparation (FASP) was conducted to digest the protein as described previously [59]. Trypsin solution was added to the protein samples at a 50:1 ratio (*w/w*), and then digested at 37 °C overnight. The digested products were desalted on C18 Cartridges (Empore^TM^ SPE Cartridges C18, Sigma, St. Louis, MI, USA), and then lyophilized and redissolved with 40 μL of dissolution buffer. Finally, the peptide content was determined by ultra-violet light spectral density at 280 nm. The peptides (100 μL) were collected from each sample for iTRAQ labelling by AB SCIEX nanoLC-MS/MS (Triple TOF 6600) [60].

### 4.7. Peptide Fractionation with Strong Cation Exchange (SCX) Chromatography

The iTRAQ-labelled peptides were mixed and fractionated using the AKTA Purifier 100 system as described previously [57,58], with minor modifications. Specifically, buffer solution A (10 mM KH_2_PO_4_ in 25% of ACN, pH 3.0) was added to the mixture and eluted at a flow rate of 1 mL/min with 0% buffer solution B (0.5 M KCl, 10 mM KH_2_PO_4_ in 25% of ACN, pH 3.0) for 25 min; then, 0–8% buffer solution B was eluted from 25 to 32 min, 10–20% buffer solution B from 32 to 42 min, 20–45% buffer solution B from 42 to 47 min, 45–100% buffer solution B from 47 to 52 min, and 100% buffer solution B from 52 to 60 min. Buffer solution B was reset to 0% at 60 min. The elution was monitored by absorbance at 214 nm, and fractions were collected once every minute, and then desalted on C18 Cartridges (Empore^TM^ SPE Cartridges C18, Sigma, St. Louis, MI, USA).

### 4.8. Liquid Chromatography-Tandem Mass Spectrometry (LC-MS/MS) Data Analysis, Protein Identification, and Quantification

Each sample was separated using the Easy nLC HPLC system as described previously [57,58]. The peptide mixture was loaded onto a sample column equilibrated with 0.1% buffer A (formic acid), and then separated through the analytical column (Thermo Scientific Easy Column) at a flow rate of 300 μL/min.

The MS/MS analysis was performed using the Q-Exactive mass spectrometer in the positive ion mode. The scanned area was set at *m/z* 300–1800, the automatic gain control target was set to 3 × 10^6^, the maximum injection time was 50 ms, and the dynamic exclusion duration was 60 s. Twenty MS2 scans were obtained after every scan. The MS2 activation type was set as HCD spectra with a resolution of 17,500 at *m/z* 200 and an isolation width at *m/z* 2. The normalized collision energy was 30 eV and the underfill ratio was defined as 0.1%. The instrument was run with the peptide recognition mode enabled [57,58]. Finally, the RAW file from the MS analysis was loaded to Mascot 2.2 (Matrix Science, London, UK; version 2.2) and Proteome Discoverer 4.1 (Thermo Electron, San Jose, CA, USA) for protein identification and quantification, based on the sesame protein database (assembly S_indicum_v1.0, 35,410 protein sequences, https://www.ncbi.nlm.nih.gov/genome/?term=sesamum), using the search parameters described previously [61]. The significance level was defined as *p* < 0.05 for identifying differentially expressed proteins (DEPs) for further analyses [62,63].

### 4.9. Gene Ontology (GO) Annotation

The DEP sequences were retrieved in batches from the UniProtKB database in FASTA format. The retrieved sequences were locally searched against the SwissProt database using the National Center for Biotechnology Information basic local alignment search tool (BLAST)+ client software (ncbi-blast-2.2.28+-win32.exe) to search for homologous sequences for the transfer of the functional annotations to the studied sequences. The top 10 blast hits with E-values less than 1 × 10^−3^ for each queried sequence were retrieved and loaded into Blast2GO 3.3.5 (http://www.geneontology.org) for GO mapping and annotation [64]. An annotation configuration with an E-value filter of 1 × 10^−6^, default gradual EC weights, a GO weight of 5, and an annotation cut-off of 75 were chosen. Unannotated sequences were then annotated with more permissive parameters. The sequences without BLAST hits and unannotated sequences were then used for an analysis with InterProScan against the EBI databases to retrieve functional annotations of protein motifs and merge the InterProScan GO terms with the annotation set [65].

### 4.10. Kyoto Encyclopedia of Genes and Genomes (KEGG) Pathway Annotation

The FASTA sequences of the DEPs were blasted against the online KEGG database (http://geneontology.org/) to retrieve their KEGG orthology entries (KOs). These were mapped to the KEGG pathways [66] and were extracted for further analyses.

### 4.11. Functional Enrichment Analysis

To further explore the function of the DEPs on cell physiological processes and to identify the relationships between the DEPs, an enrichment analysis was performed. The GO enrichment (including biological process, molecular function, and cellular component) and KEGG pathway enrichment analyses were applied based on Fisher’s exact test, considering all quantified protein annotations as the background dataset. Benjamini–Hochberg correction for multiple testings was further applied to adjust the derived *p*-values. Only the functional categories and pathways with *p*-values under 0.05 were considered significant.

### 4.12. Quantitative Real-Time PCR (q-PCR) Assays

After treatment of the RNA samples with RNase-free DNase I, the total RNA was used as the template for cDNA synthesis using the PrimeScript™ RT reagent Kit with gDNA Eraser (Perfect Real Time) (TaKaRa, Shiga, Japan). The q-PCR assays for the detection of relative gene expression were performed using SYBR Premix Ex Taq II (Tli RNaseH Plus) (TaKaRa, Shiga, Japan) with the Applied Biosystems StepOne Plus Real-time PCR System (Thermo Fisher Scientific). The thermal cycle conditions were as follows: 95 °C for 3 min, followed by 40 cycles at 95 °C for 10 s and 60 °C for 30 s. A melting curve analysis was conducted to ensure primer specificity as follows: 95 °C for 15 s, 60 °C for 1 min, 60–95 °C for 15 s (+0.3 °C per cycle). The expression data were normalized using the public reference gene *AtActin2* [67] and the relative gene expression was calculated using the 2^−ΔΔCT^ method [68]. The gene-specific primers for the q-PCR assays are listed in Table S1.

### 4.13. Statistical Analyses

Significant differences (*p*-value < 0.05) were determined using the one-way analysis of variance, followed by Tukey’s honestly significant difference (HSD) multiple comparison tests, using Statistical Package for the Social Sciences 17.0 (SPSS, Chicago, IL, USA).

## 5. Conclusions

In this study, we investigated the physiological differences in the WT and *nla* mutant in response to N limitation, and they were further investigated by integrated transcriptional and proteomic profiling analyses. The early-senescence of the *nla* mutant resulted from excess N remobilization. It has been reported that NLA targets NRT1.7 and regulates NO_3_^−^ remobilization under N-limited conditions. Here, we found that NLA might target LHT1 and regulate organic N remobilization from source to sink under N limitation.

## Figures and Tables

**Table 1:** Genes involved in photosynthesis, protein synthesis and degradation in the WT and *nla* mutant under N-limited conditions ^a.^

Gene ID	Description	WT		*nla*	
		Fold Change	*p*-Value	Fold Change	*p*-Value
**Photosynthesis**					
At1g55670	subunit G of photosystem I	−1.90	0.002	−4.32	0.003
At1g49380	cytochrome c biogenesis protein family	−2.10	0.002	−8.01	0.005
At5g11450	23 kDa polypeptide of water-oxidizing complex of photosystem II	−2.18	0.003	−3.64	0.005
At3g48730	glutamate-1-semialdehyde 2,1-aminomutase 2 (GSA 2)	−2.25	0.003	−3.63	0.003
At1g03130	photosystem I reaction center subunit II	−2.41	0.002	−4.53	0.005
At4g28660	photosystem II reaction center W (PsbW) family protein	−2.53	0.004	−5.08	0.002
At3g26060	peroxiredoxin Q	−2.71	0.003	−7.15	0.002
At3g14930	uroporphyrinogen decarboxylase	−3.42	0.001	−2.75	0.005
At2g40490	uroporphyrinogen decarboxylase	−3.51	0.002	−3.11	0.002
At4g18480	magnesium-chelatase subunit chlI	−3.74	0.002	−4.90	0.003
**Protein synthesis**					
At1g74970	ribosomal protein S9	−1.99	0.002	−4.85	0.003
At2g38140	chloroplast 30S ribosomal protein S31	−2.03	0.003	−4.16	0.004
At1g32990	ribosomal protein L11 family protein	−2.11	0.004	−6.02	0.002
At5g13510	ribosomal protein L10 family protein	−2.18	0.003	−4.72	0.002
At5g27820	ribosomal protein L18 family protein	−2.25	0.002	−3.16	0.001
At5g47190	ribosomal protein L19 family protein	−2.26	0.003	−5.64	0.004
At1g79850	chloroplast 30S ribosomal protein S17	−2.36	0.002	−5.52	0.001
At4g29060	elongation factor Ts family protein	−2.52	0.003	−4.68	0.004
At3g15190	chloroplast 30S ribosomal protein S20	−2.53	0.002	−7.40	0.003
At2g24090	ribosomal protein L35 family protein	−2.54	0.001	−6.40	0.003
At4g24770	RNA-binding protein cp31	−2.59	0.001	−5.50	0.004
At3g52380	RNA-binding protein cp33	−2.78	0.003	−4.46	0.003
At2g33450	chloroplast 50S ribosomal protein L28	−3.27	0.001	−5.32	0.001
At3g13120	chloroplast 30S ribosomal protein S10	−3.75	0.002	−6.71	0.003
At2g33450	chloroplast 50S ribosomal protein L28	−4.20	0.001	−7.45	0.001
At3g08740	elongation factor P (EF-P) family protein,	−4.87	0.001	−5.12	0.001
**Proteolytic degradation**					
At3g57680	peptidase S41 family protein	7.48	0.002	5.82	0.005
At5g37540	aspartyl protease	4.23	0.002	13.36	0.005
At5g13800	hydrolase, alpha/beta fold family	4.15	0.001	5.86	0.003
At2g05630	autophagy protein APG8d (AtAPG8d)	3.87	0.001	3.91	0.002
At4g21980	autophagy protein APG8a (AtAPG8a)	3.13	0.001	5.27	0.001
At4g04620	autophagy protein APG8b (AtAPG8b)	2.90	0.003	4.52	0.005
At4g01610	cathepsin B-like cysteine protease	2.79	0.001	5.11	0.002
At5g51070	ATP-dependent Clp protease ATP-binding subunit (ClpD)	2.19	0.004	4.27	0.003
At1g11910	aspartyl protease	2.13	0.003	2.20	0.005
At3g15580	autophagy protein APG8i (AtAPG8i)	2.04	0.003	2.26	0.002

^a^ Fold change was calculated by comparing the transcription abundance in the plants grown under N-limited conditions with the plants grown under N-sufficient condition.

**Table 2:** Comparison of the genes involved in N metabolism and transport, senescence, and anthocyanin synthesis in the WT and *nla* mutant under N-limited conditions ^a.^

Gene ID	Description	WT		*nla*	
		Fold Change	*p*-Value	Fold Change	*p*-Value
**Nitrogen transport**					
At5g40780	lysine and histidine specific transporter (AtLHT1)	NC		18.65	0.001
At4g35180	amino acid transporter family protein (AtLHT7)	NC		15.02	0.001
At4g21120	cationic amino acid transporter (AtCAT1)	NC		6.21	0.002
At2g38290	high-affinity ammonium transporter 2 (AMT2)	NC		3.75	0.004
At1g31830	amino acid permease family protein (AtPUT2/AtPQR2)	NC		2.93	0.002
At5g63850	amino acid transporter 4 (AAP4)	NC		2.55	0.002
At3g56200	amino acid transporter	NC		2.36	0.001
At4g13510	ammonium transporter 1 (AMT1.1)	NC		2.22	0.004
At1g58360	neutral amino acid transporter (AtAAP1)	NC		2.07	0.002
**Genes involved in anthocyanin synthesis**					
At2g37040	phenylalanine ammonia lyase (PAL1)	9.00	0.002	4.03	0.003
At2g30490	cinnamic acid 4-hydroxylase	5.41	0.003	4.23	0.001
At5g13930	chalcone synthase (CHS)	7.61	0.004	2.28	0.002
At3g51240	flavanone 3-hydroxylase (F3H)	22.64	0.003	4.98	0.005
At5g42800	dihydroflavonol 4-reductase (DFR)	31.11	0.005	8.47	0.004
At4g22880	anthocyanidin synthase (ANS)	49.24	0.005	8.85	0.005
At4g22870	anthocyanidin synthase (ANS)	33.89	0.003	7.43	0.004
At3g21560	UDP-glycosyltransferase	8.48	0.001	3.82	0.005
At5g17050	glycosyltransferase family	10.65	0.003	NS	
At3g53260	phenylalanine ammonia-lyase (PAL2)	3.47	0.003	NC	
At1g65060	4-coumaroyl-CoA synthase 3 (4CL3)	9.50	0.001	NS	
At5g05270	chalcone-flavanone isomerase family	17.38	0.004	NS	
At1g66390	MYB domain containing transcription factor (MYB90)	29.11	0.005	35.98	0.005
At2g47190	myb family transcription factor (MYB2)	NC		24.55	0.001
At1g56650	MYB domain containing transcription factor (MYB75)	12.70	0.004	NS^c^	
At4g34990	myb family transcription factor (MYB32)	3.60	0.002	NC	
**Senescence-associated genes**					
At2g19190	senescence-responsive receptor-like serine/threonine kinase (SIRK)	NC		9.56	0.005
At5g45890	senescence-specific SAG12 protein (SAG12)	NC		62.49	0.004
At5g14930	leaf senescence-associated protein (SAG101)	NC		6.54	0.002
At5g66170	senescence-associated family protein	NC		6.26	0.001
At3g10980	senescence-associated protein.	NC		2.37	0.004

^a^ Fold change was calculated by comparing the transcription abundance in the plants grown in the N-limited conditions with the plants grown under N-sufficient conditions. ^b^ NC represents no change. ^c^ NS represents no significance.

**Table 3:** Differentially expressed proteins involving photosynthesis and chlorophyll biosynthesis and organization pathways in the *nla* vs. WT comparison under N limited conditions ^a.^

Protein ID	Description	Fold Change	*p*-Value
**Photosynthesis**			
P56780	Photosystem II reaction center protein H	−2.46	0
Q8LC58	Photosystem I reaction center subunit IV B, chloroplast (PSI-E B)	−2.16	0
Q9SA56	Photosystem I reaction center subunit II−2, chloroplastic	−1.93	0.01
A0A178W7I8	PSAG	−1.88	0.003
P10796	Ribulose bisphosphate carboxylase small chain 1B, chloroplastic	−1.84	0.004
Q8LCA1	Protein CURVATURE THYLAKOID 1B, chloroplastic	−1.78	0.003
Q8VY52	PsbP domain-containing protein 2, chloroplastic	−1.77	0.001
Q39195	Photosystem II 5 kDa protein, chloroplastic	−1.72	0.001
A0A178WK60	Chlorophyll a-b binding protein, chloroplastic	−1.69	0.001
Q9XF88	Chlorophyll a-b binding protein CP29.2, chloroplastic	−1.68	0
A0A178UXI3	Photosystem II reaction center Psb28 protein	−1.66	0.008
A8MS75	Chlorophyll a-b binding protein, chloroplastic	−1.64	0.001
P49107	Photosystem I reaction center subunit N, chloroplastic	−1.63	0
Q8H112	PGR5-like protein 1A, chloroplastic	−1.62	0
Q9SY97	Photosystem I chlorophyll a/b-binding protein 3-1, chloroplastic	−1.55	0
Q9SYX1	Light-harvesting complex-like protein 3 isotype 1, chloroplastic	−1.54	0
Q9FPI3	Chlorophyll a-b binding protein, chloroplastic	−1.54	0.032
P82538	PsbP-like protein 1, chloroplastic	−1.53	0.001
Q9S7W1	Chlorophyll a-b binding protein CP29.3, chloroplastic	−1.51	0.003
**Chlorophyll biosynthesis**			
**and organization**			
Q9SKT0	Protein THYLAKOID FORMATION 1, chloroplastic	−1.96	0
Q9M591	Magnesium-protoporphyrin IX monomethyl ester [oxidative] cyclase, chloroplastic	−1.5	0.002

^a^ Fold change was calculated by comparing the abundance of the proteins in the *nla* mutant with that in WT.

**Table 4:** Differentially expressed proteins involving in protein synthesis, protein degradation, and N metabolism and transport in the *nla* vs. WT comparison under N limited conditions ^a.^

Protein ID	Description	Fold Change	*p*-Value
**Protein synthesis and folding**			
Q9LJE4	Chaperonin 60 subunit beta 2, chloroplastic	−1.90	0.001
A0A178VU06	Peptidyl-prolyl cis-trans isomerase	−1.65	0.001
Q9SMQ9	DnaJ-like protein	−1.62	0.001
A0A1P8ART2	Molecular chaperone Hsp40/DnaJ family protein	−1.59	0.004
P21240	Chaperonin 60 subunit beta 1, chloroplastic	−1.51	0.002
A0A178VZ96	Peptidyl-prolyl cis-trans isomerase	−1.51	0.000
**Proteolysis**			
F4HX35	Autophagy-related protein	1.61	0.005
Q9T075	Protein RMD5 homolog	2.09	0.000
**Ubiquitination pathway**			
Q42202	Ubiquitin-60S ribosomal protein L40-2	−2.14	0.023
Q9CA23	Ubiquitin-fold modifier 1	−1.68	0.044
P59232	Ubiquitin-40S ribosomal protein S27a-2	−1.61	0.005
P25865	Ubiquitin-conjugating enzyme E2 1	−1.52	0.004
**Nitrogen metabolism and transport**			
Q9M390	Protein NRT1/PTR FAMILY 8.1	1.67	0.003
P11832	Nitrate reductase [NADH] 1	1.81	0.008
O04907	Nitrilase 2	1.88	0.030
A0A178UFA7	LHT1	2.09	0.003
Q9FGS5	High-affinity nitrate transporter 3.1	2.15	0.027

^a^ Fold change was calculated by comparing the abundance of the proteins in the *nla* mutant with that in WT.

**Table 5:** The identical members from transcriptional and proteome analysis.

Gene ID	Protein ID	Description	Fold Change^a^	*p*-Value^a^	Fold Change^b^	*p*-Value^b^
AT3G12500	P19171	Basic endochitinase B	3.15	0.01	3.74	0.00
At4g37430	Q8H137	Putative cytochrome P450 monooxygenase (CYP91A2)	2.67	0.00	5.20	0.00
At1g59710	A0A1P8AQI0	Actin cross-linking protein (DUF569)	2.54	0.01	3.48	0.00
AT2G43510	Q42328	Defensin-like protein 195	2.33	0.04	44.29	0.00
At4g16260	A0A1P8B3U2	Glycosyl hydrolase superfamily protein	2.28	0.01	16.58	0.00
AT1G21250	Q39191	Wall-associated receptor kinase 1	2.20	0.00	4.43	0.00
AT5G40780	A0A178UFA7	LHT1	2.09	0.00	18.65	0.00
AT1G02920	Q9SRY5	Glutathione S-transferase F7	2.02	0.01	13.55	0.00
At2g16710	A8MR92	Iron-sulfur cluster biosynthesis family protein	2.00	0.00	3.51	0.00
At5g27760	A0A1P8BCY5	Hypoxia-responsive family protein	1.87	0.03	4.57	0.00
At2g37130	F4IQ05	Peroxidase	1.86	0.04	4.35	0.00
AT1G59820	Q9XIE6	Phospholipid-transporting ATPase 3	1.85	0.00	2.67	0.00
AT1G70690	Q8GUJ2	Plasmodesmata-located protein 5	1.82	0.01	8.92	0.00
At5g15870	Q9LFT3	Glycosyl hydrolase family 81 protein	1.77	0.02	2.65	0.00
AT1G11310	A0A1P8AMJ7	MLO-like protein	1.76	0.00	3.55	0.00
AT3G48090	B2BDD6	Enhanced disease susceptibility 1	1.73	0.02	4.86	0.00
AT4G32690	Q67XG0	Two-on-two hemoglobin-3	1.72	0.00	2.29	0.00
AT5G04930	P98204	Phospholipid-transporting ATPase 1	1.71	0.00	3.29	0.00
At3g15810	Q9LVZ8	Protein LURP-one-related 12	1.69	0.01	2.52	0.00
AT1G76150	Q8VYI3	Enoyl-CoA hydratase 2, peroxisomal	1.64	0.01	4.48	0.00
AT1G20630	Q96528	Catalase-1	1.63	0.00	7.12	0.00
At5g16450	Q9FFE0	Putative 4-hydroxy-4-methyl-2-oxoglutarate aldolase 2	1.63	0.01	3.12	0.00
At3g05230	Q9MA96	Signal peptidase complex subunit 3A	1.61	0.00	3.76	0.00
AT4G08850	Q8VZG8	MDIS1-interacting receptor like kinase 2	1.56	0.00	6.29	0.00
AT4G28390	O49447	ADP,ATP carrier protein 3, mitochondrial	1.55	0.00	5.61	0.00
AT3G17790	Q9SCX8	Purple acid phosphatase 17	1.52	0.01	7.23	0.00
AT1G17840	Q8RXN0	ABC transporter G family member 11	1.51	0.03	0.43	0.00
AT4G16760	O65202	Peroxisomal acyl-coenzyme A oxidase 1	1.50	0.03	4.60	0.00
At1g63010	Q2V4F9	SPX domain-containing membrane protein At1g63010	1.50	0.01	2.47	0.00
AT1G23740	Q9ZUC1	NADPH-dependent alkenal/one oxidoreductase, chloroplastic	0.66	0.00	0.43	0.00
At5g27560	A0A1R7T377	DUF1995 domain protein, putative (DUF1995)	0.66	0.00	0.32	0.00
At3g47070	Q94CB6	Uncharacterized protein At3g47070	0.65	0.00	0.25	0.00
AT5G02120	O81208	Light-harvesting complex-like protein OHP1, chloroplastic	0.64	0.00	0.30	0.00
AT5G43750	Q9FG89	Photosynthetic NDH subunit of subcomplex B 5, chloroplastic	0.63	0.01	0.09	0.00
At4g21210	B9DHI2	AT4G21210 protein	0.63	0.00	0.32	0.00
AT1G10370	Q9FUS7	Glutathione S-transferase	0.62	0.04	0.34	0.00
AT4G22890	Q8H112	PGR5-like protein 1A, chloroplastic	0.62	0.00	0.39	0.00
AT1G70410	Q94CE4	Beta carbonic anhydrase 4	0.61	0.00	0.19	0.00
AT1G64750	Q9XIR8	Protein DELETION OF SUV3 SUPPRESSOR 1(I)	0.61	0.00	2.31	0.00
At1g74730	Q94F10	Transmembrane protein, putative (DUF1118)	0.61	0.00	0.43	0.00
At3g61870	F4IX01	Plant/protein	0.60	0.00	0.20	0.00
AT5G07440	O82179	Glycine cleavage system H protein 2, mitochondrial	0.60	0.00	11.46	0.00
AT3G07390	Q94BT2	Auxin-induced in root cultures protein 12	0.59	0.00	5.75	0.00
At4g33500	Q93V88	Probable protein phosphatase 2C 62	0.59	0.01	0.39	0.00
AT2G23670	O64835	At2g23670/F26B6.32	0.57	0.02	0.31	0.00
AT2G26540	A0A1P8AZL4	Uroporphyrinogen-III synthase family protein	0.57	0.00	0.34	0.00
AT5G66570	P23321	Oxygen-evolving enhancer protein 1-1, chloroplastic	0.56	0.00	0.35	0.00
At5g52780	Q9LTD9	Uncharacterized protein PAM68-like	0.53	0.01	0.38	0.00
AT2G20890	Q9SKT0	Protein THYLAKOID FORMATION 1, chloroplastic	0.51	0.00	0.23	0.00

^a^ Data from proteome analysis. ^b^ Data from transcriptome analysis.

## Supplementary Materials

Supplementary Materials can be found at https://www.mdpi.com/1422-0067/21/6/2171/s1. Table S1. Primers of the genes targeted in the q-PCR assays. Figure S1. Identification and analysis of proteome in WT and the *nla* mutant. Figure S2. Peptide ion score distribution of the detected peptides.

## Abbreviations

CLC	Chloride channel
DEP	Differentially expressed protein
GO	Gene Ontology
GOGAT	Glutamine synthetase/glutamate synthase
KEGG	Kyoto Encyclopedia of Genes and Genomes
KO	KEGG orthology
NLA	N limitation adaption
NO_3_^−^	Nitrate
NT	Nitrate reductase
NRT	NO_3_^−^ transporter
NUE	N use efficiency
Pi	Inorganic phosphate
q-PCR	Quantitative real-time PCR
WT	Wild-type
