# Supplementary material for: Integrated Transcriptional and Proteomic Profiling Reveals Potential Amino Acid Transporters Targeted by Nitrogen Limitation Adaptation
Source: Int J Mol Sci. 2020 Mar 21;21(6):2171. doi: 10.3390/ijms21062171 (PMC7139695; doi:10.3390/ijms21062171)
Supplement: Supplementary file 1 [file ijms-21-02171-s001.pdf]

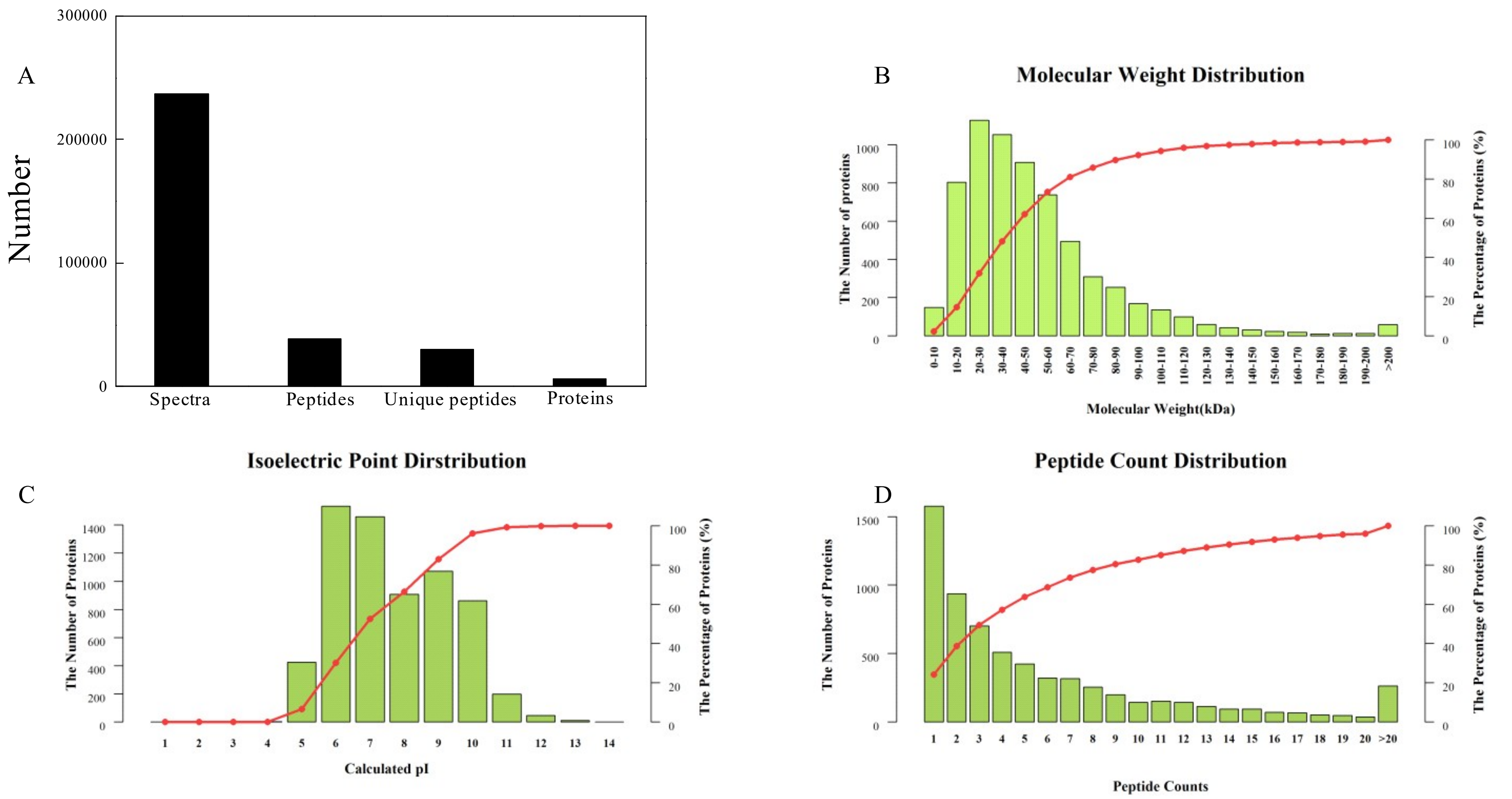

Fig S1. Identification and analysis of proteome in WT and the *nla* mutant. (A)Total spectra, peptides, unique peptides and identified proteins by iTRAQ proteomic analysis. (B) The molecular distribution,(C) Isoelectric point distribution, (D) Peptide count distribution.

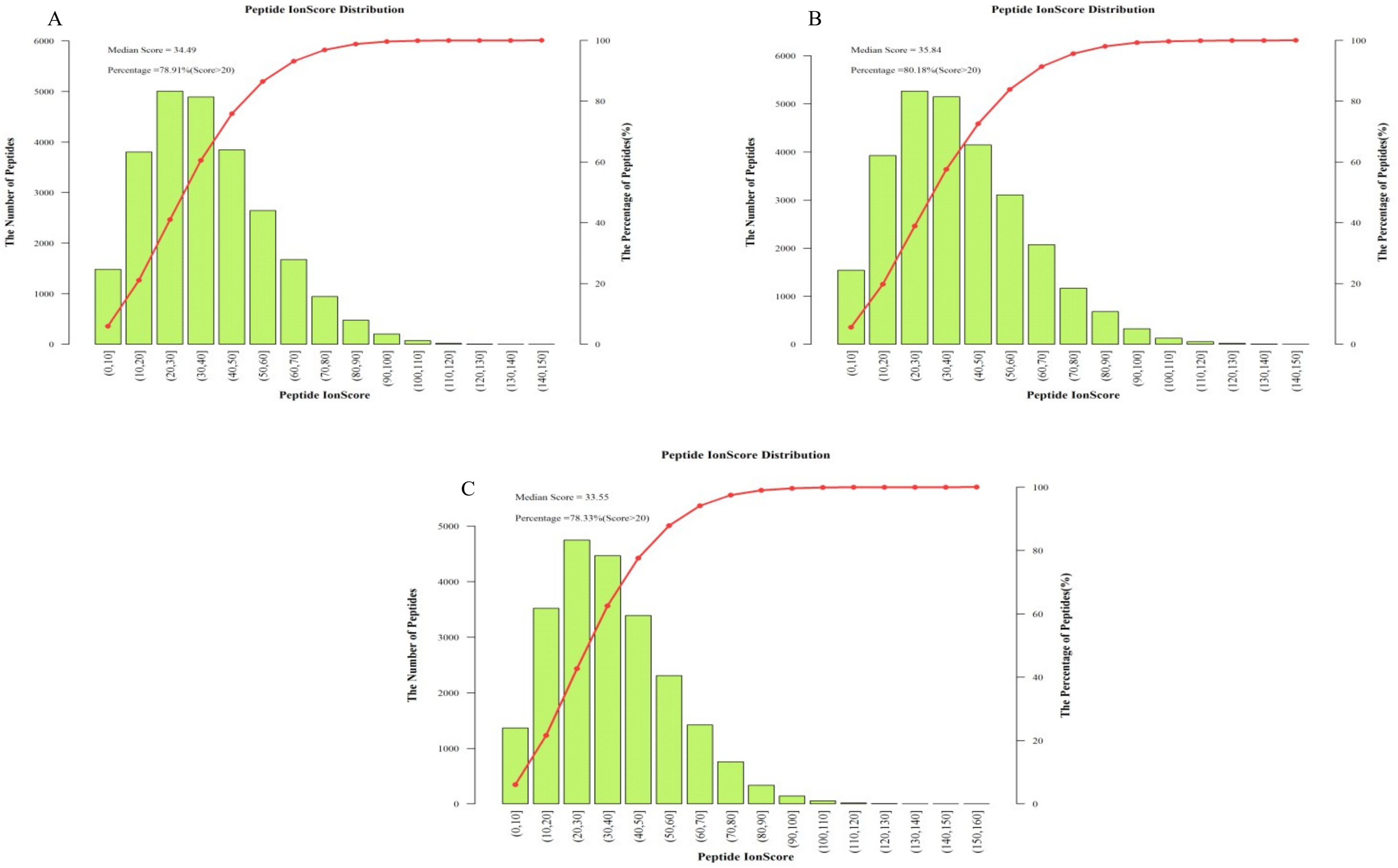

Fig S2. Peptide ionscore distribution of the detected peptides.

Table S1 The primers of the genes for q-PCR assays

Genes	Forward primer (5' to 3')	Reverse primer (5' to 3')
AtActin2	AGTGGTCGTACAACCGGTATTGT	GATGGCATGAGGAAGAGAGAAAC
AtMYB2	CAAACTCTCCAACACATGAAG	CTCTTCTAACATCTGGACGTAAG
AtNRT1.7	GCAACAGCCAGGACCCACTCAG	AACCCCAAAAGGGATGCTACAA
AtLHT1	AGTCATCGTTGCTTACATCGTCGT	TGGCGATAGGACCATCAAGAAAAGA
AtORE1	CTTACCATGGAAGGCTAAGATGGG	TCGGGTATTTCCGGTCTCTCAC
AtRNS3	AAGCTGGTCTCAAGCTCAAACAG	TCCGGTTTGATCCCAGCATTGG
AtSAG29	TAAGCGCCGTTATGTGGTTCGC	ATCCCACCACGTTTGGAATCGC
AtVIN2	CAAAGGCAAACCACCTCATGGC	TCTGAGTGGGACCCATAGAACTCG
